# Phenotypic, Molecular and Symbiotic Characterization of the Rhizobial Symbionts of *Desmanthus paspalaceus* (Lindm.) Burkart That Grow in the Province of Santa Fe, Argentina

**DOI:** 10.1371/journal.pone.0104636

**Published:** 2014-08-25

**Authors:** Laura Viviana Fornasero, María Florencia Del Papa, José Luis López, Francisco Javier Albicoro, Juan Marcelo Zabala, María Antonieta Toniutti, José Francisco Pensiero, Antonio Lagares

**Affiliations:** 1 IBBM - Instituto de Biotecnología y Biología Molecular, CONICET - Departamento de Ciencias Biológicas, Facultad de Ciencias Exactas, Universidad Nacional de La Plata, La Plata, Argentina; 2 Facultad de Ciencias Agrarias, Universidad Nacional del Litoral, Santa Fe, Argentina; Rochester Institute of Technology, United States of America

## Abstract

*Desmanthus paspalaceus* (Lindm.) Burkart belongs to the *D. virgatus* complex, subfamily Mimosoidae. The known potential as livestock fodder of several of these legumes prompted us to undertake a phenotypic, molecular, and symbiotic characterization of the *D. paspalaceus* symbionts in the Santa Fe province, Argentina. The rhizobia collected—containing isolates with different abiotic-stress tolerances—showed a remarkable genetic diversity by PCR fingerprinting, with 11 different amplification profiles present among 20 isolates. In selected isolates 16S-rDNA sequencing detected mesorhizobia (60%) and rhizobia (40%) within the collection, in contrast to the genus of the original inoculant strain CB3126—previously isolated from *Leucaena leucocephala*—that we typified here through its 16S rDNA as *Sinorhizobium terangae*. The results revealed the establishment by diverse bacterial genera -rhizobia, sinorhizobia, and mesorhizobia- of full N_2_-fixing symbiotic associations with *D. paspalaceus*. This diversity was paralleled by the presence of at least two different *nodC* allelic variants. The identical *nodC* alleles of the *Mesorhizobia* sp. 10.L.4.2 and 10.L.5.3 notably failed to group within any of the currently described rhizo-/brady-/azorhizobial *nodC* clades. Interestingly, the *nodC* from *S. terangae* CB3126 clustered close to homologs from common bean nodulating rhizobia, but not with the *nodC* from *S. terangae* WSM1721 that nodulates *Acacia*. No previous data were available on *nod*-gene phylogeny for *Desmanthus* symbionts. A field assay indicated that inoculation of *D. paspalaceus* with the local *Rhizobium* sp. 10L.11.4 produced higher aerial-plant dry weights compared to *S. teranga* CB3126–inoculated plants. Neither the mesorhizobia 10.L.4.2 or 10.L.5.3 nor the rhizobium 10L.11.4 induced root nodules in *L. leucocephala* or *P. vulgaris*. The results show that some of the local isolates have remarkable tolerances to several abiotic stresses including acidity, salt, and temperature; while exhibiting prominent N_2_ fixation; thus indicating suitability as candidates for inoculation of *D. paspalaceus*.

## Introduction

The family Leguminosae comprises more than 700 plant genera and nearly 19,000 species, including annual herbs and woody perennials, with a distribution over a broad range of ecologic conditions [1], [2]. The geographically wide and environmentally diverse distribution of legumes, together with their capacity to establish N_2_-fixing symbiosis with rhizobia [3], have made this plant family and their bacterial symbionts the focus of intensive investigation to improve the N-fertility of soils. The available information indicates that, out of all the legume species, only a portion (about 20% [4]) have been examined for nodulation and shown to have the ability to fix atmospheric N_2_. For this reason and the potential of the rhizobia-legume symbioses has prompted a survey and evaluation of new rhizobial-legume systems with an aim at developing new sustainable agricultural practices.

*Desmanthus* is a genus of the subfamily Mimosoidae of the family Leguminosae; with the latter constituting a highly polymorphic group whose members exhibit a very wide range of characteristics, with several species being suitable for animal feeding [5]–[7]. The genus *Desmanthus* includes about 24 species native of America [8]–[10]. From a taxonomic point of view, six of these species (*D. acuminatus*, *D. glandulosus*, *D. paspalaceus*, *D. pubescens*, *D. tatuhyenis* and *D. virgatus*) integrate the *Desmanthus virgatus* complex, with distribution to the Greater and Lesser Antilles, Trinidad and Tobago, and the Galapagos Islands and to the Americas from Texas to Argentina [8]–[10]. The species has been planted throughout the tropics and subtropics and has probably become naturalized in many other areas worldwide (http://www.fs.fed.us/global/iitf/pdf/shrubs/Desmanthus%20virgatus.pdf). In Argentina, *D. virgatus* complex comprises *D. paspalaceus*, *D. acuminatus*, *D. virgatus* and *D. tatuhyensis*, with the first three having potential forage value [11], [12]. The available data show that the crude protein content of whole plants ranges from 10.5 to 15.5%, with the leaves containing 22.4% and the stems 7.1%, and that the crop yields approach the 35 tons/ha/year of dry matter [10]. The *Desmanthus* potential as fodder for livestock [5], [6], [13] prompted the early release of several cultivars in Australia [14] as well as in the USA [13]. The evaluations of *D. virgatus* have indicated a unique role for this legume in extensively managed pastures on neutral to alkaline clay soils, where few other legumes can adapt [15].

In Argentina, the three species of the *D. virgatus* complex with potential as forage are present; and recently, Zabala et al. [16] have reported the collection of new germplasm, the subsequent analysis of the existing morphologic diversity, and the use of numerical methods to characterize variation patterns. As an indication of the value of the local germplasm, several accessions of *D. virgatus*, *D. paspalaceus*, and *D. acuminatus* evaluated in other countries—including the cv. Marc that produces high quality and palatable forage—were collected in Argentina [14]. The known ability of wild species of the *D. virgatus* complex to tolerate seasonal drought and severe competition from grass and herbs points to members of this legume complex as constituting a promising alternative for animal feeding in semiarid regions of north central and northwest Argentina [16].

Several investigations have focused on the isolation, analysis, and symbiotic characterization of rhizobial strains that are able to associate with *Desmanthus* spp. [17]–[21]. On the basis of earlier studies, Australians have employed rhizobia isolated from *Leucaena leucocephala* and *Neptunia plena* as field inoculants [17], with variable plant responses depending on the soil, field site, and the titer of indigenous rhizobia present. Thus, inoculation of *D. virgatus* with the *Rhizobium* strain CB3126 was reported to increase plant-leaf production relative to the uninoculated controls both in greenhouses [19] and in the field [20]. Strain CB3126 proved to nodulate and fix nitrogen in different *D. virgatus* accessions and cultivars and had thus been recommended early on as an inoculant for this legume [14], [17]. The response to those inoculations, however, occurred in soils where only few rhizobia or none at all were present [19]. Since strain CB3126 had been originally isolated from the *L. leucocephala* [17], restrictions in symbiotic competitiveness and efficiency on *Desmanthus* could have occurred since the inoculation was with a strain recovered from a heterologous host plant [22]. Thus, because of the forage potential of several *Desmanthus* spp. [14], [23], [24], the need arose to investigate the nature and symbiotic characteristics of the rhizobia that associated with these legumes at the diversity of locations in North, Central, and South America where those species were found. With *D. illinoensis* in particular, the pioneering work of Beyhaut et al. [21] had characterized the symbiosis and phylogeny of the root-nodule isolates collected in the USA. That most of the isolates were identified as *Rhizobium giardinii*, with only two of them being more closely related to *R. etli* (originally reported as *R. leguminosarum*), was notable. Though none of these isolates from *D. illinoensis* were infective on *D. virgatus*—thus evidencing a plant-species–specific requirement for the nodulation of *Desmanthus* spp. [21]—; Sinsuwongwat et al. reported later the presence of *D. virgatus* plants in Thailand nodulated by *R. leguminosarum* [25]. To date, the microsymbionts of *Desmanthus* spp. grown in South America, and particularly in Argentina, have not yet been systematically studied.

We report here the isolation, genetic diversity, phenotypic characterization, and phylogenetic analysis of rhizobia that nodulate *D. paspalaceus* (member of *D. virgatus* complex) and that were isolated from soil samples of the Santa Fe province in central Argentina. The bacteria collected included isolates having a broad range of tolerance to different abiotic stresses and a remarkable genetic diversity, with *Mesorhizobium* being reported by the first time among the nitrogen-fixing symbionts of *D. paspalaceus*.

## Materials and Methods

### Population studied of *D. paspalaceus*

Seeds of *D. paspalaceus* were collected in a field of Villa Guillermina, Department of General Obligado, Province of Santa Fe, Argentina at the coordinates 28.0°18.0′30.0″W, 59.0°16.0′29.3″S (Eastern-humid Chaco region) [16]; and identified under number Pensiero 6932 in the SF Herbarium Facultad de Ciencias Agrarias, Universidad Nacional del Litoral, Santa Fe, Argentina. Seeds were collected from 20–40 plants to ensure a representative sample, and were stored at room temperature until the time of planting.

### Cultivation and preservation of rhizobia

Yeast extract-mannitol (YEM) medium [26] and TY medium [27] were used for the routine cultivation of rhizobia (at 28°C). The rhizobia isolated from *D. paspalaceus* root nodules were preserved in agar-containing YEM, and in 20% (v/v) glycerol stocks at −20°C. For solid media, 15 g of agar per liter of medium were added.

### Establishment of a collection of *D. paspalaceus*-nodulating rhizobia isolated from soil samples collected in Santa Fe, Argentina

The seeds collected in the field were used to generate trapping plants to recover *D. paspalaceus*-nodulating rhizobia from a soil sample obtained at the same location where the original seeds had been harvested. Nodules collected either from plants grown in pots with vermiculate and soil [1∶1 ratio] or from the plants removed from the field were surface-sterilized 8 min. in 30 vol. H_2_O_2_, washed with distilled water, and crushed in 100 µl of Fåhraeus [28] mineral solution. Only isolates 10C1.1 and 10C1.8 were recovered from nodules collected in the field. Bacterial clones isolated from this suspension in YEM or TY agar plates were confirmed in their *D. paspalaceus*–nodulation phenotype and preserved for further studies at −20°C in 20% (v/v) glycerol-TY.

### Evaluation of rhizobial tolerance to abiotic stresses operating in the Argentine soils populated with *D. paspalaceus*

Rhizobia isolated from the root nodules of *D. paspalaceus* (*cf.* below) were evaluated in their capacity to grow in YEM solid media under different stressing conditions. To evaluate the osmotic tolerance of the isolates 10 µl of a culture dilution containing *ca.* 10^4^ cells were spotted onto YEM plates with increasing concentrations of NaCl—*i. e.*, 0.5%, 1%, 2%, and 3% (w/v). The ability of each isolate to grow under the different concentrations of NaCl was recorded after a 5-day incubation of the plates at 28°C. In a similar assay, the pH tolerance of the isolates was investigated with YEM plates containing solid media at pHs adjusted to 4.0, 5.0, 6.0, 7.0, 8.0, 9.0, and 10.0. Finally, the effect of temperature on the rhizobial growth was analyzed with YEM plates (pH 7.0) incubated at 28°C, 35°C, 40°C, or 45°C. In all of these assays the bacterial growth was estimated on a scale of 0 to 5 (0, the absence of growth; 5, full development).

### Nodulation tests in vermiculate-mineral medium for evaluating the ability of selected rhizobial isolates to support plant growth in the absence of fixed nitrogen

*D. paspalaceus* seeds collected in the field were multiplied under green house conditions. For plant tests seeds were boiled 10 sec. in distilled water for coat disruption [29]. Then, seeds were surface sterilized using 96% ethanol (1 min.), and 3% NaClO (5 min.) [30]. Seeds were finally rinsed with sterile distilled water and kept in water one hour for swelling. The seeds were then germinated in 1% (w/v) water-agar plates and the seedlings individually planted in plastic pots containing vermiculite and Jensen mineral solution [31]. To investigate the ability of selected rhizobial strains to support plant growth in the absence of fixed nitrogen, 15-day old plants were inoculated with 10 ml of a rhizobial suspension containing *ca.* 10^7^ c.f.u./ml in Jensen mineral medium. Each strain was evaluated at least in triplicate, with uninoculated and N-fertilized plants serving as controls. The N-fertilization was performed by adding to each pot 50 ml of 0.05% (w/v) KNO_3_ (70 ppm N) weekly during the 65 days, resulting in *ca.* 30 mg N incorporated into each pot with its single plant. The experiment was carried out in a plant-growth chamber at 28°C with a 16 h-light photoperiod. After harvesting, the dry weights of the aerial part and of the root were determined for each plant. The data were subjected to an analysis of variance and an F test. When significant differences were detected the results from the different treatment protocols were compared by Tukey's test.

### Field testing of rhizobia

The trial was conducted in the Experimental Area of the School of Agricultural Sciences of the Universidad Nacional del Litoral, located in the town of Esperanza (31°25′S, 60°56′W), Department Las Colonias, Province of Santa Fe, Argentina. In such location the field assay did not require any specific permission, and did not involve endangered or protected species. The trial was conducted during the second half of January 2012 and lasted through the middle of the following May (*i. e.*, from summer through the middle of autumn). The site has an average annual temperature of 18.8°C, and an average annual rainfall of 1,046 mm. The soil is a Typic Argiudoll with silt loam of the following chemical characteristics: 2.4% organic matter [32], 0.15% total nitrogen (Kjeldahl), 25 p.p.m. available phosphorus [33], pH 6.3 (soil∶water ratio, 1∶2.5), and 0.7 dS/m electrical conductivity. When soil samples from the experimental field were used to count the nodulating rhizobia by the method of the most probable number, fewer than 10^2^ rhizobia/g were found. The field assays to evaluate forage production were performed with *D. virgatus* cv. Marc from the Australian Tropical Crops and Forages Collection (Queensland Department of Primary Industries and Fisheries, Australia). The experiment was conducted as a randomized complete block design with 3 independent blocks and the following 4 treatments: I) uninoculated control plants, II) N-fertilized plants (7 applications of 20 kg N/ha as solid urea, separated 2 weeks each), III) plants originating from seeds that had been inoculated with strain CB3126 (the recommended strain for *D. virgatus*) immediately before sowing, and IV) plants originating from seeds that had been inoculated by a similar procedure with the local isolate 10L11.4. The plots (four/block) consisted of four lines one meter long and spaced 0.15 m apart. The area of each plot was 0.8 m^2^. The summer forage *Setaria italica* (moha) was sown in the alleys between the plots within the same block, at a density of 20 kg/ha, to reduce the rhizobial cross-contamination. The inoculants were prepared in sterile peat (*ca.* 10^9^ c.f.u./g, with rhizobia previously grown in YEM) and used at a proportion of *ca.* 8 g of inoculant/kg of seeds (with 20% [w/v] sterile sucrose as adherent), to give a final inoculum of *ca.* 10^4^ c.f.u./scarified seed (of either strain CB3126 or the isolate 10L11.4). The linear seed density at sowing was *ca.* 100 seeds/m. The final proportion of plant establishment was near 15%. Plants from the central strip of each parcel were harvested 15 weeks after sowing and dried at 60°C for dry-weight estimation. Statistical analysis was performed with the software package InfoStat 2008 (FCA, Universidad Nacional de Córdoba, Argentina). The analysis of variance (ANOVA) and Tukey's test were performed on both the plant dry weights and the total N per plant for the comparison of the treatments.

### DNA preparation and manipulation

Small-scale preparations of total DNAs were carried out according to Meade et al. [34]. All DNA manipulations were performed by previously established techniques [35].

### PCR reactions, oligonucleotide primers, and cycling conditions used

#### (i) BOXA1R PCR genomic-fingerprinting analysis

Total DNA-amplification fingerprints were performed with the BOXA1R primer as previously described by Versalovic et al. [36] with the following minor modifications stated in brief: PCR mixtures of 25 µl contained 50 mM Tris (pH 8.3), 3 mM MgCl_2_, 200 mM deoxynucleoside triphosphates, 1 U of *Taq* polymerase (Promega Corp.), and a 10-mM concentration of primer BOXA1R. The cycling conditions were as follows: 94°C for 7 min followed by 30 cycles at 94°C for 10 s, at 52°C for 1 min, and at 72°C for 2 min. After the reaction, 10 µl of the PCR products were separated in 1% (w/v) agarose gels containing 0.5 to 1 µg of ethidium bromide per ml.

#### (ii) Amplification of partial sequences of the nodC genes

Fragments of 848 bp from the *nodC* gene from isolates that nodulated *D. paspalaceus* were amplified with the primers nodCF–nodCI and the cycling conditions previously described by Laguerre et al. [37]. Alignments were performed using sequence stretches that covered *ca.* 753 bp with the homolog positions to nucleotides 353 to 1105 in the *S. meliloti* 1021 *nodC* (GI:14523570).

#### (iii) Amplification of a partial sequence of the 16S rDNA and PCR genotyping of isolates by means of specific primers

DNA fragments of *ca.* 1,440 bp containing partial nucleotide sequences of the 16S rDNAs present in isolates that had nodulated *D. paspalaceus* were amplified by means of the primers fD1 and rD1 described by Weisburg et al. [38]. Alignments were performed using sequence stretches that covered the homolog positions to nucleotides 91 to 1364 in the *S. meliloti* 1021-16S rDNA (NC_003047). Based on the 16S-rDNA sequences of the *Mesorhizobium* sp. 10.L.4.2 and 10.L.5.3 and the homologous sequence of the *Rhizobium* 10.L.11.4 (the three isolates characterized in this report) the following specific primers and PCRs were designed: For PCR1, primer 16S4 (5′-GGTTACCAGAAATGGTTTCC-3′) and primer rD1 were used to amplify an internal 538-bp fragment of the 16S rDNA of the mesorhizobia; while for PCR2, primers fD1 and 16S2 (5′-ATGGAAGAGGTGAGTGGAAT-3′) were used to amplify the homologous internal rDNA fragment from the rhizobia.

### DNA sequencing, sequence treatment, and phylogenetic analysis

The nucleotide sequences of the *nodC* and 16S-rDNA PCR products were obtained at the sequencing service from INTA Castelar, Argentina. The final sequences were deposited in GenBank under the accession numbers KJ128392 to KJ128398. DNA- and protein-similarity searches were carried out with the BLAST program from the National Center for Biotechnology Information [39]. Nucleotide sequences were aligned with ClustalW (Thompson *et al.*, 1994). Phylogenetic analyses were performed through the use of the MEGA 5.05 software package and the setting indicated in each specific sample.

### Statistical calculations and analyses

General statistical calculations were performed by means of the software package InfoStat 2008 (FCA, Universidad Nacional de Córdoba, Argentina). For the principal-components analysis (PCA) of the stress-tolerance phenotypes the XLSTAT software package was used.

## Results

### Isolation of rhizobia from root nodules of *D. paspalaceus* collected in the field and cultivated under laboratory conditions. Analysis of the rhizobial diversity by genomic-DNA fingerprinting

**Figure 1:**
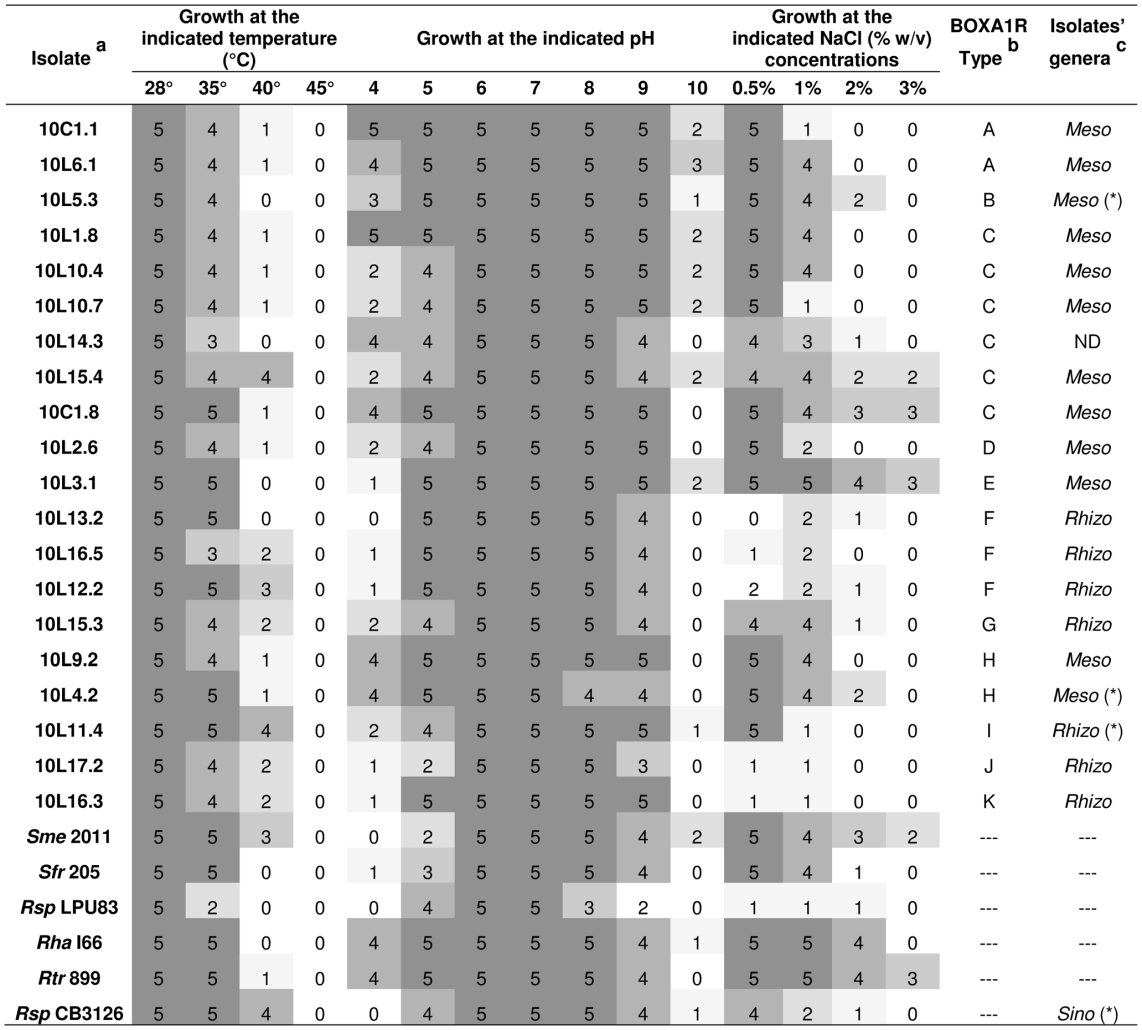
Growth of rhizobia that nodulate *Desmanthus virgatus* evaluated under different abiotic stressing conditions in agarized-YEM medium. Scores from 5 to 0 indicate the ability of rhizobia to grow under the investigated condition (5 = full development in 2 days, 0 = absence of growth). **a:** *Sme, Sinorhizobium meliloti; Sfr, S. fredii; Rsp., Rhiizobium sp.; Rha, R. hainanense; Rtr, R. tropici*. **b:** BOXA1R types according to the genomic fingerprints shown in Fig. 2. **c:** Genus of the isolate deduced from the PCR 1 and PCR 2 used to amplify a partial sequence of the 16S rDNA from the type isolates 10L.4.2/10L.5.4 (*Mesorhizobium* sp.) and 10L.11.4 (*Rhizobium* sp.), respectively (*cf.* Materials and Methods). For the type isolates (*) a 1,440-bp fragment from their 16S rDNA was PCR-amplified with primers fD1 and rD1 and sequenced (*cf.* Materials and Methods). *Meso*: *Mesorhizobium* (PCR-1–positive), *Rhizo*: *Rhizobium* (PCR-2–positive), ND: negative for PCR 1 and PCR 2.

**Figure 2:**
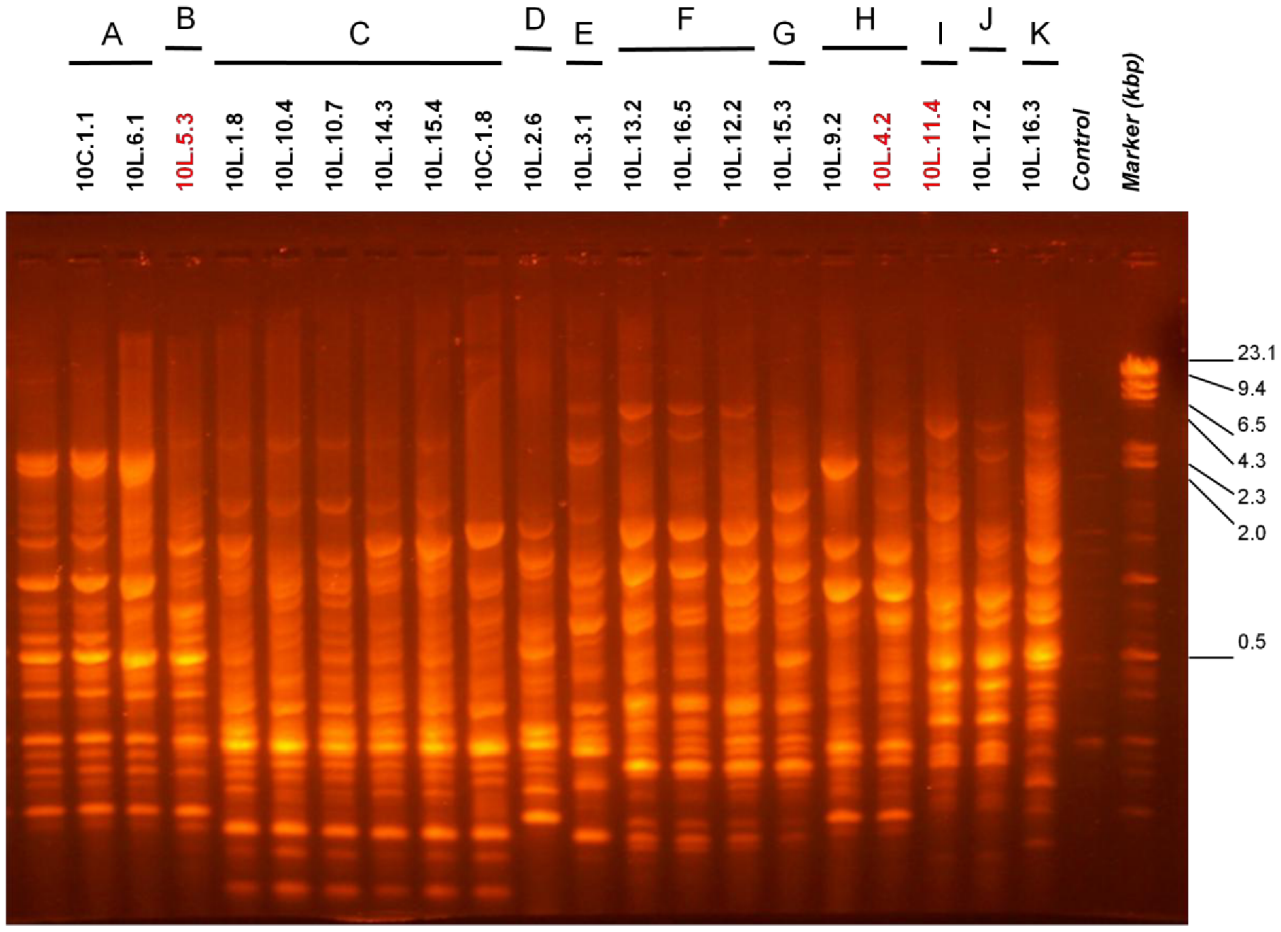
BOXA1R-PCR–fingerprint patterns of the bacterial isolates from Santa Fe (Argentina) recovered from *D. paspalaceus* root nodules (both field and laboratory isolates). The figure shows the BOXA1R-PCR products obtained with total DNA from the indicated isolates. Control lane: no template DNA added to the PCR mixture. Isolates were ordered in the gel according to the similarity of their amplification patterns and then classified in BOXA1R types as demarcated above the figure. The molecular weights of the standard DNAs (in base pairs) are indicated on the right.

*D. paspalaceus* plants grown in the laboratory in pots with vermiculate and soil were used to trap their associated root-nodule rhizobia. Only two isolates were obtained from root nodules recovered directly from one of the *D. paspalaceus* plants removed from the field. The collection of rhizobia comprised a total of 20 isolates listed in Fig. 1 (left column), including those recovered from a total of 15 different trapping plants grown in pots (*i. e.*, 18 isolates) plus the 2 from a single plant that had been nodulated in the field. In order to investigate the genetic heterogeneity of the rhizobial germplasm associated with *D. paspalaceus*, a genomic BOXA1R-PCR–fingerprinting analysis was performed with the 20 isolates. Fig. 2 shows the results of this analysis, where at least 11 different amplification patterns can be distinguished (from “A” to “K” in the figure), thus evidencing a remarkable genetic diversity among the rhizobia present in the soil sampled from Santa Fe that are able to nodulate *D. paspalaceus*. The genetic heterogeneity of the rhizobial collection was also observed when we used ERIC PCR [36], [40] to perform the analysis (not shown) in order to confirm the diversity of the collected rhizobial germplasm.

### Phenotypic diversity of the isolated rhizobia including the expression of tolerance to abiotic stresses that operate in local agricultural soils

**Figure 3:**
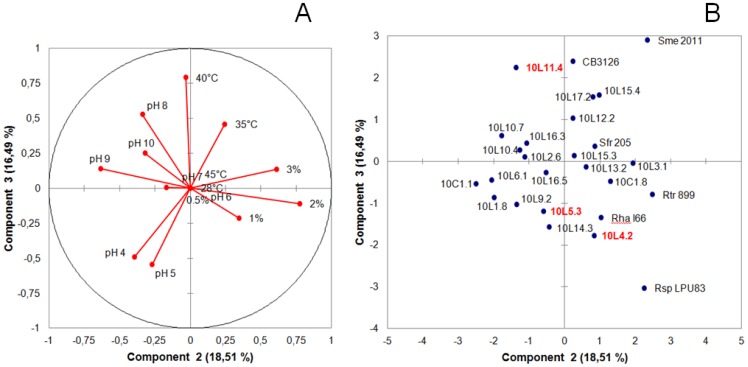
Principal-components-analysis–based separation of isolates according to their differences in tolerance to abiotic stresses. **A:** Vector-correlation plot between the variables examined (*i. e.*, stress tolerances) and the components of variation PC2 and PC3. **B:** Isolate distribution in the PC2 and PC3 spaces of variation. The PCA analysis (Pearson-n; XLSTAT software package) was performed with respect to the numerical-tolerance ranking of each isolate for each stress (variables) listed in Fig. 1. The distribution of isolates in the PC2–PC3 space (representing more than 32% of the total variation) served to separate isolates into regions of clear dominance with respect to each of the investigated stress-tolerance phenotypes: lower left, tolerance to low pH; upper left, tolerance to high pH; upper center, temperature tolerance; right center, salt tolerance. The isolates in red correspond to those selected for further molecular and symbiotic analysis (*cf.* Figs.  4–7).

In order to explore the phenotypic diversity in our collection of isolates, the rhizobia were evaluated for their capacity to grow in YEM solid media under the different stress conditions (*e. g.*, high temperature, extreme pH, high salt) that, to varying extents, operate in agricultural soils (Fig. 1). In TY liquid medium at 28°C the isolates behaved as fast growing rhizobia (*cf.* the growth curves of selected isolates in the supplementary Fig. S1). A PCA (*P*rincipal *C*omponent *A*nalysis) of the results from Fig. 1 showed that several (and in some instances all) isolates of each of the more abundant BOXA1R types A, C, and F clustered, respectively, at three different regions in the two-dimensional space of components 1 and 2 (PC1 vs. PC2, Fig. S2). These two components together represented more than 50% of the observed phenotypic variation (*cf.* areas in red, blue, and green in the supplementary Fig. S2). Moreover, since the PCA components 2 and 3, together representing nearly 35% of the phenotypic variation, were found to be strongly influenced by the pH, salt, and temperature tolerance of the isolates (Fig. 3), the inspection of these components facilitated an identification of these three phenotypes in the collection of rhizobia presented in Fig. 1. Though most isolates were able to grow in the presence of a moderate amount of salt (0.5% [w/v] NaCl), isolate 10L3.1 was the only one able to grow well in YEM medium supplemented with 2% (w/v) NaCl (score 4, in scale 0–5)(isolate on the right side of Fig. 3B). This isolate, however, had a poor growth at pH 4.0. Most isolates showed significant growth over a wide range of pH, with isolates 10L1.8, 10C1.1—and to a lesser extent 10L6.1, 10L9.2, and 10C1.8—being able to grow normally within the range of pH 5 to pH 9 (*cf.* their location in Fig. 3B). That isolates 10L.4.2 and 10L.5.3 exhibited the combined ability to grow well between pH 5 and 9 and in 1% (score 4)–2% (score 2) NaCl as well was of particular interest. Moreover, that both isolate 10L11.4 and the reference strain CB3126 were able to grow at 40°C, a temperature frequently found in the geographic area populated by the *D. paspalaceus* in Argentina, was also remarkable. The results indicated that the rhizobial collection from Fig. 1 included isolates with different degrees of tolerance to abiotic stresses, with certain ones—such as 10L4.2 and 10L5.3—being tolerant to more than a single stress (Fig. 1). As such, those rhizobia constituted suitable candidates for further evaluation of their symbiotic properties.

### The taxonomic position of chromosomal and symbiotic markers from rhizobia associated with *D. paspalaceus* plants growing in Santa Fe, Argentina

#### 16S rDNA

**Figure 4:**
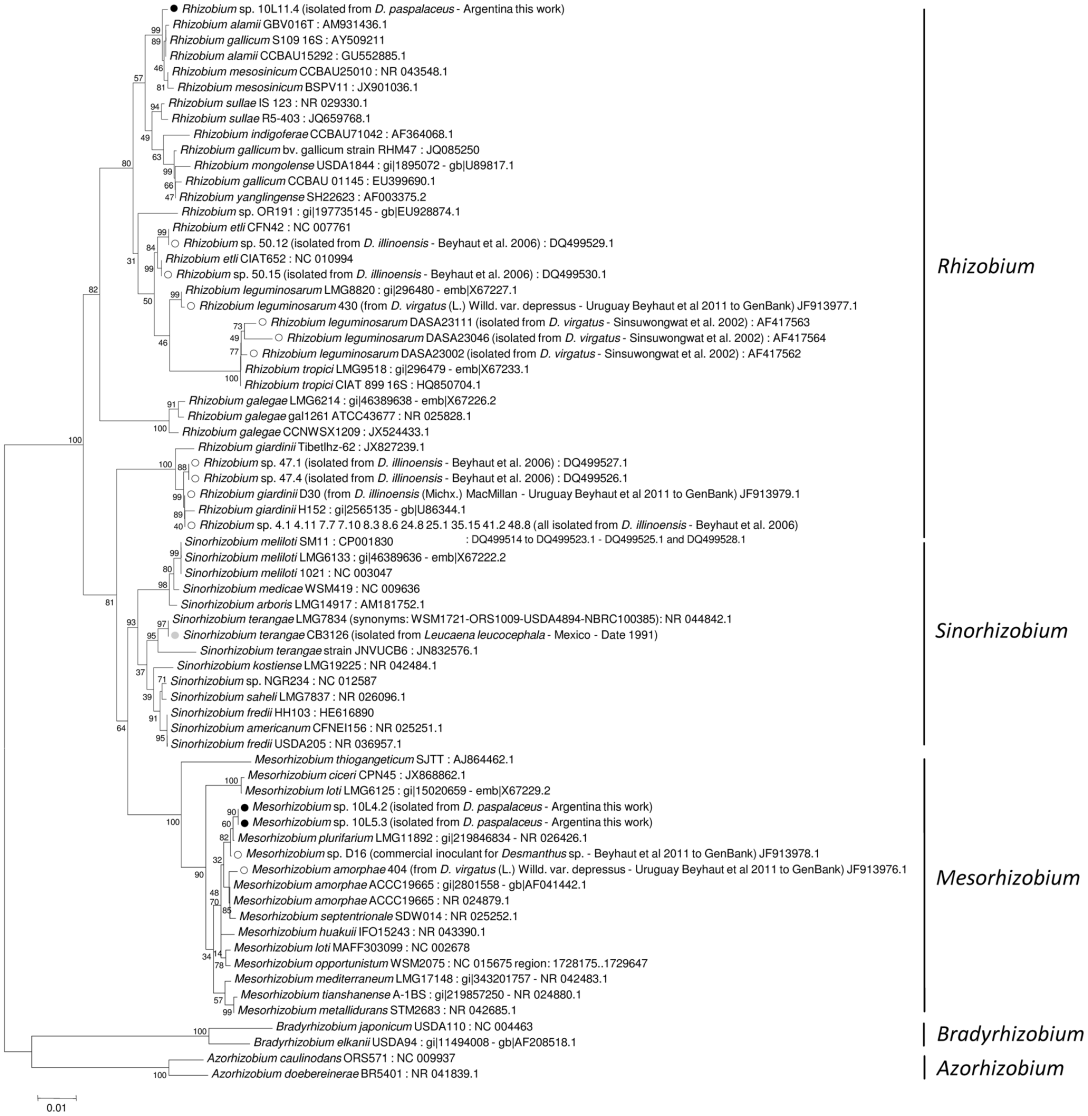
Phylogenetic (neighbor-joining) tree showing the relationship between rhizobia based on 16S-rDNA sequences including the isolates recovered from *D. Paspalaceus* plants collected in Santa Fe, Argentina. The rDNA sequences were analyzed by the neighbor-joining method through the use of the MEGA 5.05 software package. Alignments were performed using sequence stretches that covered the homolog positions to nucleotides 91 to 1364 in the *S. meliloti* 1021-16S rDNA (NC_003047)(*cf.* Materials and Methods). The bootstrap-consensus tree inferred from 1,000 replicates was taken to represent the evolutionary history of the genes (and the corresponding taxa) analyzed [57]. The percentage of replicate trees in which the associated taxa clustered together in the bootstrap test (1,000 replicates) is shown next to the branches. The tree is drawn to scale, with branch lengths in the same units as those of the evolutionary distances used to infer the phylogenetic tree. The evolutionary distances were computed by means of the Poisson-correction method [58], where the units are the number of nucleotide substitutions per site. The DNA sequences used were obtained from GenBank under the accession numbers indicated after the name of each rhizobium. Black circles (•) indicate isolates characterized in this work, open circles (o) denote rhizobia that nodulated the indicated *Desmanthus* species, and gray circles (•) correspond to the inoculant strain CB3126 isolated from *Leucaena leucocephala*.

A portion of the 16S ribosomal DNA (rDNA) was amplified by PCR following the experimental conditions described in Materials and Methods. The nucleotide sequence of the amplification products of isolates 10L.4.2, 10L.5.3, 10L.11.4 along with that of the reference strain CB3126 were analyzed in order to infer their taxonomic position by comparison with homologous amplicons from known rhizobia. Fig. 4 shows a 16S phylogenetic (neighbor-joining) tree, where isolates 10L.4.2 and 10L.5.3 are included within the mesorhizobial clade (close to *M. plurifarium*) and isolate 10L.11.4. within a rhizobial clade close to *R. alamii*, *R. mesosinicum*, and *R. gallicum*. According to the 16S-rDNA sequencing the reference strain CB3126 could be identified as *Sinorhizobium teranga* since the 1,437-bp 16S rDNA analyzed showed 100% identity to that of strain *S. terangae* LMG 7834. Beyhaut at al. [21] had previously reported that *R. giardinii* and *R. leguminosarum*-related rhizobia were able to nodulate *Desmanthus illinoensis*. Our results now demonstrate that the population of *D. paspalaceus* present in Santa Fe (Argentina) can be efficiently nodulated by rhizobia, mesorhizobia, and sinorhizobia (Fig. 4). Neither the mesorhizobia 10.L.4.2 or 10.L.5.3 nor the rhizobium 10L.11.4 presented in this work were, however, able to nodulate *L. leucocephala* or *P. vulgaris*. The use of specific PCR reactions to detect sequences of the mesorhizobial or rhizobial 16S rDNA (Fig. 1, last column on the right side) revealed that *ca.* 60% of the isolates were mesorhizobia, including the strains that belonged to the most abundant “type C” BOXA1R genomic group.

#### *nodC*

**Figure 5:**
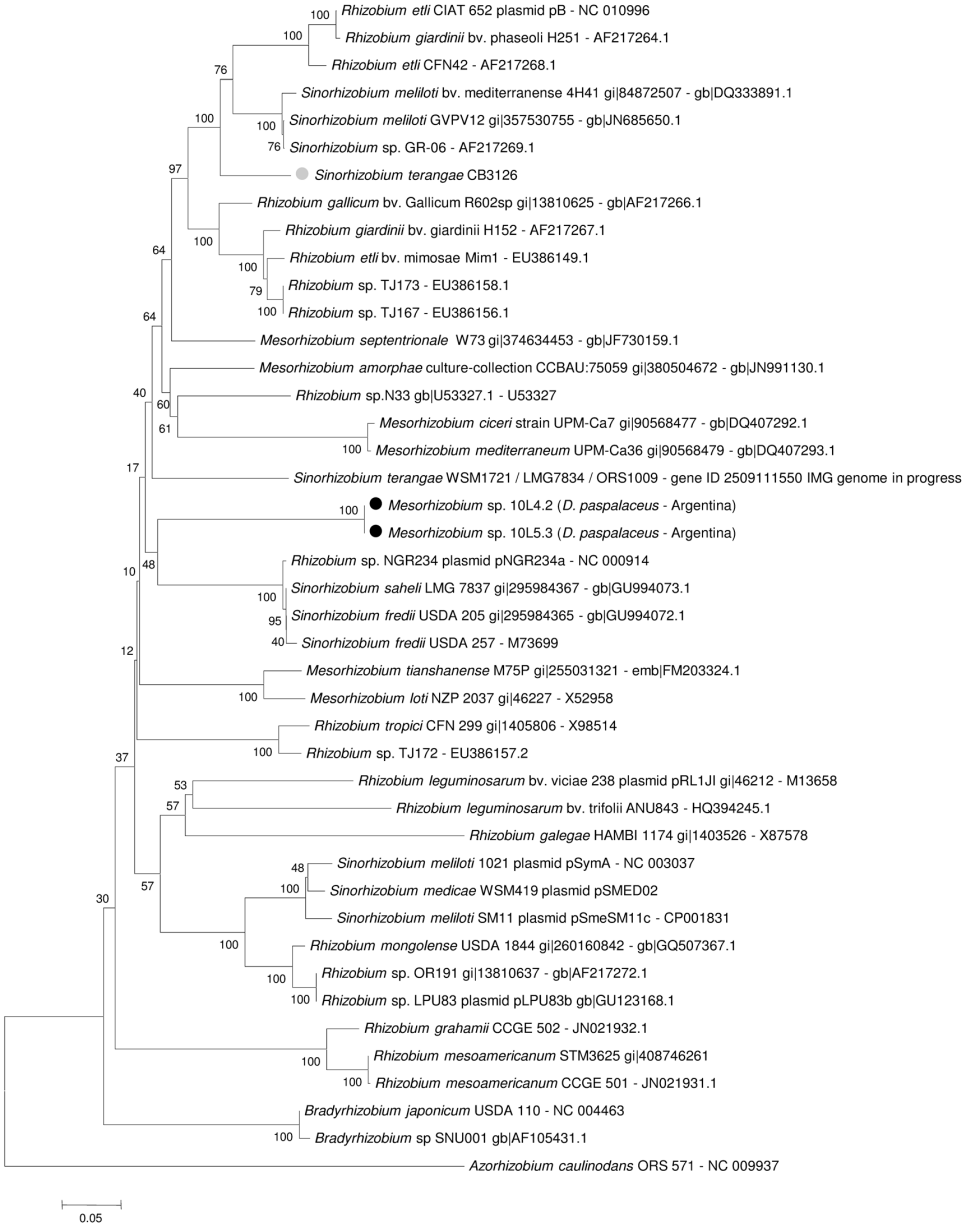
Phylogenetic (neighbor-joining) tree showing the relationship between rhizobial-nodC sequences with the inclusion of those from the isolates recovered from *D. paspalaceus* plants collected in Santa Fe, Argentina. Alignments were performed using sequence stretches that covered *ca.* 753 bp with the homolog positions to nucleotides 353 to 1105 in the *S. meliloti* 1021 *nodC* (GI:14523570). The *nodC* sequences were analyzed by the neighbor-joining method through the use of the MEGA 5.05 software package. The phylogeny was performed with a partial sequence of the *nodC* genes (*cf.* Materials and Methods and Laguerre et al. [37]). The bootstrap-consensus tree inferred from 1,000 replicates is taken to represent the evolutionary history of the genes (and the corresponding taxa) analyzed [57]. The percentage of replicate trees in which the associated taxa clustered together in the bootstrap test (1,000 replicates) is shown next to the branches. The tree is drawn to scale, with branch lengths in the same units as those of the evolutionary distances used to infer the phylogenetic tree. The evolutionary distances were computed by means of the Poisson-correction method [58], where the units are the number of nucleotide substitutions per site. The DNA sequences used were obtained from GenBank under the accession numbers indicated after the name of each rhizobium.

None of the previous studies with the nitrogen-fixing symbionts of *Desmanthus* have focused on the analysis of the *nod* genes. Thus, based on our results indicating that diverse species of rhizobia are able to nodulate *Desmanthus* (*cf.* the previous section), we investigated the *nodC* genotypes present in our isolates 10L.4.2 and 10L.5.3 as being representative of the local mesorhizobia of *D. paspalaceus*. We also partially sequenced the *nodC* gene of strain CB3126—that rhizobium originally recovered from root nodules of *L. leucocephala* [17] and here genotypified as *S. terangae* (*cf.* Fig. 4 and the previous section). Fig. 5 shows that the *nodC* gene from the strain *S. terangae* CB3126 clustered within a *nodC* clade that included several symbionts of *Phaseolus* (*e. g.*, *R. etli*, *R. giardinii*, *S. meliloti* bv. mediterranense) and *Mimosa* (*e. g.*, *Rhizobium* sp. TJ167 and TJ173). By contrast, the *nodC* fragment from the mesorhizobia 10L.4.2 and 10L.5.3, which are identical over the amplified 848 bp, did not cluster with any of reported rhizo-/azo-/bradyrhizobial *nodC* variants. Unfortunately, none of the primer pairs reported by Laguerre et al. [37] served to amplify the *nodC* fragment from the local *Rhizobium* sp. 10L.11.4., suggesting that this isolate carries a *nodC* allelic variant that is different from those present in the mesorhizobial and sinorhizobial symbionts characterized here. These results demonstrate that *D. paspalaceus* is nodulated by diverse rhizobia, and that the latter may bear phylogenetically diverse *nodC* genes.

### The symbiosis between *D. paspalaceus* and isolates 10L.4.2, 10L.5.3, and 10L.11.4 in vermiculite with mineral medium under laboratory conditions

**Figure 6:**
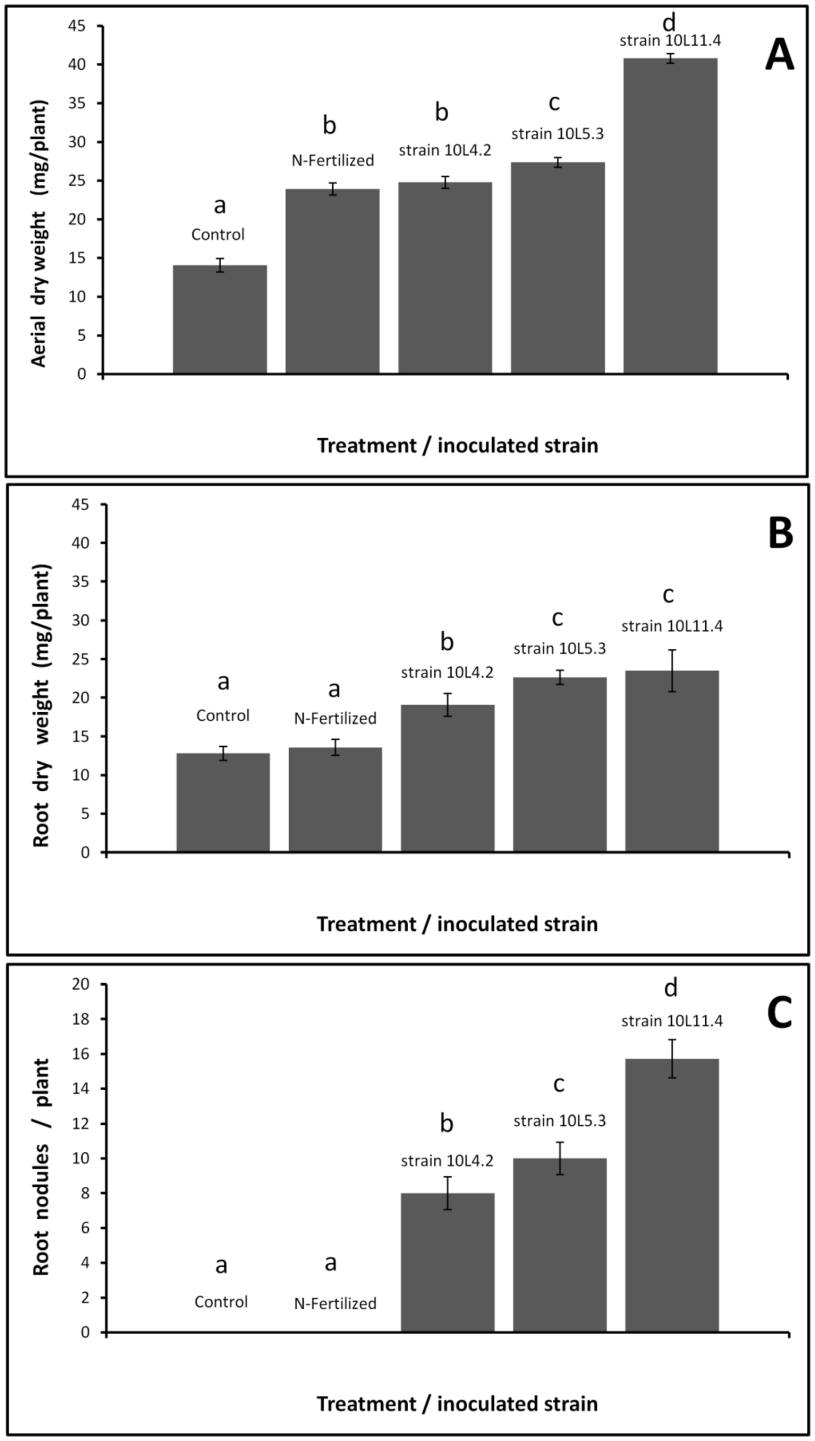
Effect of inoculant treatments on shoot dry weight per plant, root dry weight per plant, and the number of root nodules per plant, when local *D. paspalaceus* plants were grown in pots with vermiculite and mineral medium in a plant room. (A) Mean aerial weight per plant, (B) root weight per plant, and (C) number of root nodules per plant of *D. paspalaceus* plants inoculated with rhizobia and of uninoculated (control) or N-fertilized plants all grown in plastic pots with mineral medium and vermiculite in a plant room. *D. paspalaceus* plants were inoculated with rhizobia 15 days after germination as indicated in Materials and Methods. Uninoculated plants without N addition (control), and N-fertlilized plants were included in the experiment (first two bars from left to right)(see Materials and Methods). Plants were harvested 65 days after inoculation for aerial and root dry-weight analyses. The values reported in the figure are the means from 3 independent experiments including at least 3 plants each. The bars represent the standard deviation (SD). Treatments with same letters are not significantly different by Tukey's test (P<0.01).

In order to evaluate if the nodulation of *D. paspalaceus* with isolates 10L.4.2, 10L.5.3, and 10L.11.4. can support plant growth in mineral medium with vermiculite in the absence of fixed nitrogen; different plant sets were inoculated separately with each of the isolates as indicated in Materials and Methods and the shoot and root dry weights per plant analyzed at 65 days post inoculation. Fig. 6A shows that the aerial part of the plants inoculated with rhizobia all exhibited significant differences compared to the uninoculated control (p≤0.01). Furthermore, inoculation with isolates 10L.5.3 and 10L.11.4 in both instances resulted in higher aerial dry weights than that of the N-fertilized control (p≤0.01). In general, the remarkably higher dry weight per plant observed for those inoculated with isolate 10L.11.4 constituted a *ca.* 70% increase over the values measured in the N-fertilized plants and a 190% increase over the figure for the uninoculated controls. The dry weight of the aerial part of the plants positively correlated with the number of nodules induced by each rhizobial isolate (Fig. 6C), suggesting that the higher biomass observed in plants inoculated with isolate 10.L.11.4 was attributable to its higher nodulation capacity, and not necessarily only to an improved (strain-specific) nitrogen-fixing ability. These results demonstrated that plants inoculated with the local isolate 10.L.11.4 had a higher aerial biomass than the N-fertilized control plants and that the three strains tested induced an increased root biomass compared to that of the N-fertilized plants. That effect on root growth by the inoculated rhizobia could favor plant tolerance to nutritional and drought stresses by improving nutrient and water uptake through the generation of a more extensive root system.

### Symbiotic performance of isolate *Rhizobium* sp. 10L.11.4 and the strain *S. terangae* CB3126 in a field-release assay

**Figure 7:**
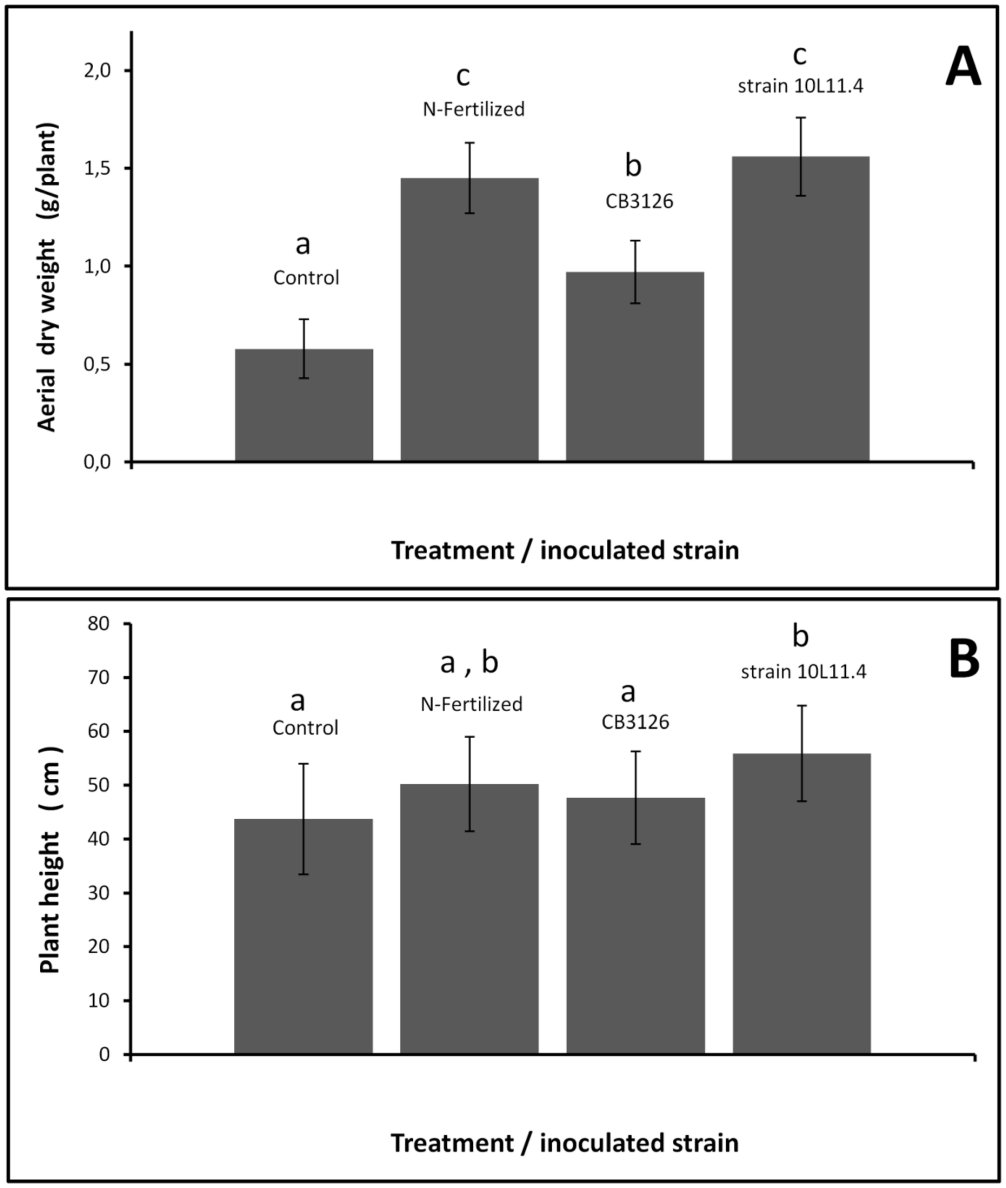
Effect of N-fertilization and seed inoculation with rhizobia on the (A) shoot dry weight and (B) plant height of *D. virgatus* plants (cv. Marc) grown in an open field in a Typic Argiudoll soil in the province of Santa Fe, Argentina. The experiment was conducted as a randomized complete block desig*n* with 3 independent blocks and four treatments as indicated in the figure. The assay was carried out in a Typic Argiudoll soil with silt-loam for ca. 4 months (112 days) in the summer–early-autumn season (February–April) in an experimental field of the Universidad Nacional del Litoral, Santa Fe, Argentina (31°25′S, 60°56′W). The environmental maximum and minimum temperatures (mean ± SD) were 28.5±5.8°C and 17.7±5.2°C, respectively. An average soil temperature of 24±3.8°C was registered, with a total rainfall of 388 mm distributed throughout the assay. Control plots in each block were sown with uninoculated seeds. The N-fertilized plot received 7 applications of 20 kg N/ha as solid urea, separated 2 weeks each during the 16 weeks of the field assay. In plots to evaluate rhizobia, no nitrogen was added, and the *D. virgatus* seeds were inoculated with each of the indicated rhizobial strains immediately before sowing as indicated in Materials and Methods. The plants from all the plots (n = ≥10–12 plants per plot) were harvested 16 weeks after sowing for analysis of their aerial dry weight. The values reported in the figure are the means over the three blocks of the assay. The bars represent the SD. Treatments with same letters are not significantly different by Tukey's test (P<0.01).

The symbioses of isolate 10L.11.4 and of the type strain CB3126 recovered from root nodules of *L. leucocephala* were both evaluated in the open field with the commercial *D. virgatus* cv. Marc as host plant. The experiment was conducted as a random blocked assay in a Typic Argiudoll soil with silt loam containing 0.15% (w/w) total N and fewer than 10^2^ rhizobia/g with the ability to nodulate *D. virgatus* (*cf.* Materials and Methods). The biomass of the aerial part of the plants whose seeds had been inoculated with either the isolate 10L.11.4 or the strain CB3126 (*ca.* 10^4^ c.f.u./seed at sowing) were compared with the uninoculated and the N-fertlized controls (Fig. 7). Plants inoculated with the local isolate 10L.11.4 had heights that varied between 40 and 65 cm and showed an average dry weight that was comparable to that of the N-fertilized plants (P<0.01). Although the average dry weight of the plants inoculated with strain CB3126 was higher than that of the uninoculated plants, that value was lower than the dry weight of either the plants inoculated with isolate 10L.11.4 or those N-fertilized. Under the field conditions assayed, the quality of the symbiosis estimated through plant dry weight indicated the local isolate 10L. 11.4 to be a more efficient inoculant for the cultivar Marc tested than strain CB3126. We have not yet investigated if this symbiont also promoted a stimulation of root growth in the field compared to that occurring with N fertilization as had been recorded with the plants grown in vermiculite under controlled greenhouse conditions.

## Discussion

We have analyzed the types of rhizobia associated with *D. paspalaceus* plants collected in the province of Santa Fe, Argentina and have characterized the phenotypic and symbiotic properties of those symbionts. The isolates proved to be genetically and phenotypically diverse. According to the genomic-PCR fingerprints those rhizobia could be grouped within 11 different BOXA1R profiles, one including 30% of the isolates (profile type C), with 7 profiles represented by only a single isolate. The tolerance to abiotic stresses such as acidity and salinity was remarkable for many of the isolates, while a few were also able to grow at as high as 40°C in agar-containing solid medium, a temperature frequently found in central and north Argentina where the *Desmanthus* spp. are present. From a practical point of view, the temperature tolerance displayed by some of the isolates would be expected to constitute a positive trait likely to favor rhizobial survival both in soil and while on the surface of inoculated seeds.

Despite the potential exhibited by *Desmanthus* spp. as a forage alternative for semiarid regions [16], [23], [41], [42], little work has been done on the specific rhizobial symbionts that associate with these legumes. First, Sinsuwongwat et al. [25] reported the isolation of *R. leguminosarum* from root the nodules of *D. virgatus* in Thailand. Then, in a study by Beyhaut et al. [21] the authors reported that rhizobia isolated from the root nodules of *D. illinoensis* in the USA could in most instances be identified as belonging to the species *R. giardinii*, with only few isolates being characterized as related to *R. etli* through 16S-rDNA analysis (unfortunately, no analysis of any of the *nod* genes was available for those isolates). We report here that while the isolates 10L4.2 and 10L5.3 clustered close to the type strain *M. plurifarium* LGM 11892 that was known to nodulate *Acacia* spp. and *L. leucocephala* [43], [44]; isolate 10L11.4 proved to be related to the previously reported *R. alamii* GBV016T (isolated from the rhizosphere of *Arabidopsis thaliana*) [45] and CCBAU15292 (isolated from *Albizia julibrissin*) [46], and to *R. mesosinicum* CCBAU25010 (isolated from *Glycine soja*) [47]. These data and the results from the literature taken together indicate that different *Desmanthus* spp. can be nodulated by strains of: a) *Rhizobium* (*e. g.*, *D. illinoensis* by *R. giardinii* and *R. leguminosarum*-related rhizobia [21]; *D. virgatus* by *R. leguminosarum* [25]; *D. paspalaceus* by the *R. alamii*- and *R. mesosinicum* -related rhizobia presented in this work), b) *Mesorhizobium* (this work and two strains reported to Genbank by Beyhaut et al. in 2011; *cf.* Fig. 4), and c) *Sinorhizobium*, as demonstrated here through the 16S-rDNA typing of strain CB3126, infective on *D. virgatus* and originally isolated from *L. leucocephala* [17]. Beside the diversity of microsymbionts infecting *Desmanthus* spp., Beyhaut et al. [21] reported that the type strains *R. giardinii* 56.6 and 35.1 recovered from *D. illinoensis* in the USA were unable to nodulate *D. virgatus*. Such strains were, otherwise, able to establish a full N_2_-fixing symbiosis with legumes that belonged to other genera—such as *L. leucocephala*, *Dalea purpurea*, *Psoralea esculenta*, and *Prosopis juliflora* [21]. A complex picture such as this one prompted us to characterize the type of *nod* variants (*i. e.*, *nodC*) present in our isolates and in the type strain CB3126 and to investigate the phylogenetic relationships with the *nodC* homologs from other rhizobia. The results summarized in Fig. 5 show that the *nodC* from *S. terangae* CB3126 is part of a clade that includes several *nodC* sequences from *Phaseolus*-nodulating rhizobia; an observation consistent with the previous evidence that other *Desmanthus* members were nodulated by *R. giardinii* [21], a species also infective in *Phaseolus* [48]. Unfortunately, no *nodC* sequences were available from the *R. giardinii* isolates recovered from *D. illinoensis* by Beyhaut et al. [21] in order to evaluate how those genes compared to the *nodC* type present in *S. terangae* CB3126. Interestingly, the *nodC* of the strain *S. terangae* CB3126 that nodulates *D. paspalaceus* is not related to the *nodC* from the *S. terangae* strain WSM1721 (synonyms LMG 7834, ORS1009) that nodulates *Acacia* [43]. The sequencing of the *nodC* variants present in strains such as *R. giardinii* 56.6 and 35.1 that nodulate *D. illinoensis* but not *D. virgatus* [21] will certainly help to elucidate whether or not such host preferences correlate with the sequence divergence among their *nodC* loci and those from the *D. paspalaceus* symbionts *Mesorhizobium* sp. 10L4.2 and 10L5.3, and *S. teranga* CB3126. Unfortunately, none of the PCR-primer pairs proposed by Laguerre et al. [37] were able to amplify the *nodC* allele of our local *Rhizobium* sp. 10L.11.4. This observation suggests the existence of a yet additional *nodC* variant associated with the symbionts of *D. paspalaceus*.

That a different *nodC* was observed in the *Mesorhizobium* sp. 10L4.2 and 10L5.3 is of interest. Their *nodC* loci exhibited no preferential relationship to any of the previously described *nodC* variants. The underlying question thus is whether or not the *D. paspalaceus* symbionts produce the same family of Nod signal molecules, or alternatively, all those rhizobia produce a different—albeit-symbiotically active—set of nodulation factors. Upon consideration that rhizobial *nod* phylogenies correlate well with the species of the host legumes [49], the significant phylogenetic distance observed here between the *nodC* variants present in the *Sinorhizobium teranga* CB3126 and in the *Mesorhizobium* sp. 10L.4.2. and 10L.5.3 (*cf.* Fig. 5) is striking. A deeper sequencing of each of the *nod* clusters, in addition to a structural elucidation of the corresponding Nod signal will be necessary to shed light on the early signaling in these symbioses.

In order to analyze the symbiosis between *D. paspalaceus* and isolates from each of the rhizobial genera identified in this work, we performed inoculation assays both in a plant room in N-free plant medium and later in the soil of an open field. The assays in the plant room demonstrated that *Rhizobium* sp. 10L11.4 and the *Mesorhizobium* spp. 10L4.2 and 10L5.3 were all able to support plant growth in the absence of a source of fixed nitrogen. The convenience of inoculating *D. virgatus* in soils with only few or no native rhizobia had been recommended by Bahnisch et al. [19]. In field assays, the maximum growth of inoculated plants compared to uninoculated controls had also been indicated as positive though dependent on different conditions that included the type of year after the sowing, the prevalence of the indigenous rhizobia, and the degree of N-fertility of each particular soil [20]. As a consequence of those studies, the inoculation of *D. virgatus* with the efficient rhizobium CB3126 was recommended. Within this context, we demonstrated here that the inoculation of *D. paspalaceus* with our local isolates in mineral medium resulted in higher plant dry weights compared to those of uninoculated and the N-fertilized plants. The inoculation of the plant pots with vermiculite allowed a systematic inspection of the root size that revealed a root-growth–promoting effect of rhizobia (with a more than 75% increase in root mass compared to roots of uninoculated or of N-fertilized plants). Though we do not know the mechanisms operating here in the promotion of the root growth, the likelihood is that the inoculated strains could have produced one or more phytohormones, as had already been observed in other fast- [50]–[53] and slow-growing [54] rhizobia. A recent report showed that the production of cytokinins by a recombinant sinorhizobium significantly improved host-plant tolerance to drought stress [55]. In addition to the effects that the rhizobial isolates studied in this work could have exerted on drought-stress tolerance in *Desmanthus*, that the significant increase in the root size in that species triggered by rhizobia could extend to the soil region accessible to the plant, thus improving plant survival under nutritional limitations, is conceivable.

In the field, the only strain that we tested, *Rhizobium* sp. 10L11.4, proved to promote a degree of plant aerial growth that was significantly higher than that observed in the N-fertilized control plants, or in plants inoculated with the recommended strain *S. terangae* CB3126. At all events, the remarkable temperature tolerance of *Rhizobium* sp. 10L11.4 as a rhizobial inoculant for a tropical legume, together with the strain's symbiotic performance in the laboratory and the field, point to this isolate as a suitable candidate for more extensive field evaluations. All these observations reinforce the relevance of using local isolates for the inoculation of *D. paspalaceus* and indicate the necessity to screen in more detail the available rhizobial collection with an aim at a selection of the best candidates for inoculation based, as previously suggested [56], on broader selective criteria.

## Supporting Information

Figure S1 **Growth curves in TY medium of the isolates 4.2, 5.3, and 11.4 (from Santa Fe, Argentina) and strain CB3126 (from Mexico) that nodulate** ***D. paspalaceus*** **.** The panels show the growth curves of the following different rhizobia: Strain *S. meliloti* 2011 (*Sm* 2011, lower panel) was included as a fast-growing reference rhizobium. The duplication times (tD) at 28°C, indicated for each rhizobium beside the linear, logarithmic-growth phase of the curve, ranged between 2 h 20 min (isolate 11.4), and 5 h 38 min (isolate 4.2). The results are representative of two independent growth curves.
(TIF)

Figure S2 **PCA-based separation of isolates in the PC1- vs.-PC2 space according to the rhizobial differences in tolerance to abiotic stresses.** The PCA analysis (Pearson-n; XLSTAT software package) was performed on the numerical ranking of tolerance to each stress for each isolate (as the independent variables) listed in Fig. 1. The distribution of the isolates in the PC1-vs.-PC2 space (representing more than 50% of the total variation) served to separate the isolates into regions according to their BOX genomic-PCR–amplification patterns (*cf.* Fig. 2): Pattern A (red), Pattern C (blue), and Pattern F (green).
(TIF)
